# Usefulness of polymerase chain reaction tests in Chagas disease studies

**DOI:** 10.3389/fpara.2024.1292143

**Published:** 2024-02-13

**Authors:** Norma Bautista-Lopez, Momar Ndao

**Affiliations:** ^1^ National Reference Center for Parasitology, Research Institute of the McGill University Center, Montreal, QC, Canada; ^2^ Department of Microbiology and Immunology, McGill University, Montreal, QC, Canada; ^3^ Division of Experimental Medicine, McGill University, Montreal, QC, Canada

**Keywords:** *Trypanosoma cruzi* (*T cruzi*), diagnostics test, Chagas disease, PCR techniques, T. cruzi screening

## Abstract

The Polymerase Chain Reaction (PCR) test is a highly sensitive, specific, and rapid diagnostic tool for Chagas disease. Chagas disease is caused by the protozoan flagellate *Trypanosoma cruzi* and is endemic to the Americas. While conventional serological methods are still used in the diagnosis of Chagas disease, they are being gradually replaced by molecular methods like PCR. PCR can detect the parasite’s DNA in blood or tissue samples from humans and animals, including asymptomatic infections and animal reservoirs. In a study conducted on a colony of New World monkeys, PCR analysis was found to be superior to conventional screening tools for trypanosome infection, although false negatives can still occur. In clinical studies, PCR has been used to assess the effectiveness of Nifurtimox and Benznidazole in treating acute and chronic Chagas patients. However, the presence of low-grade and intermittent parasitemia in peripheral blood, even in the absence of treatment, renders PCR an unreliable test for evaluating successful treatment. Based on this limiting factor, among others, we do not believe that PCR is an appropriate gold standard test for Chagas in clinical and preclinical studies. Other diagnostic methods, such as serological and biomarker tests, should be used in conjunction with PCR techniques for more accurate diagnosis of Chagas.

## Introduction

Chagas disease, caused by the hemoflagellate protozoan *Trypanosoma cruzi*, remains a challenging medical, economic, and social burden in the Americas. According to the World Health Organization (WHO), over six million individuals are infected, and 75 million are living under the daily threat of infection (WHO, 2020).

Chagas disease is characterized by two clinical phases: the acute and the chronic phases. During the acute phase, infected individuals usually exhibit high parasitemia and experience symptoms such as fever, anorexia, and tachycardia (Rangel-Gamboa et al., 2019). In the chronic phase, infected individuals may develop various conditions affecting the cardiovascular, digestive, or neurological systems (Echavarría et al., 2021).

Depending on the clinical stage, specific laboratory diagnostic tools can be employed to confirm *T. cruzi* infection. In the acute phase, direct *T. cruzi* detection can be achieved through parasitology techniques such as xenodiagnosis, or by employing molecular biology techniques. Several polymerase chain reaction (PCR) amplification-based assays have been tested to detect *T. cruzi*, with some becoming routine tests. These assays include real-time PCR (qPCR), digital droplet PCR (ddPCR) and loop-mediated isothermal amplification PCR (LAMP-PCR). During the chronic phase, parasitemia decreases and becomes intermittent, making the indirect detection of *T. cruzi* through the presence of antibodies against *T. cruzi* crucial. The most common serological techniques employed to detect specific *T. cruzi* Ig G are enzyme-linked immunosorbent assay (ELISA), complement fixation test, fluorescent antibody technique, hemagglutination test, radioimmunoprecipitation assay, and Western blot (Alonso-Padilla et al., 2019). This perspective aims to discuss the current use of PCR techniques for detecting *T. cruzi* DNA in Chagas disease studies and explore potential new directions for utilizing these techniques in endemic areas.

## PCR techniques for *T. cruzi* identification

Traditional parasitological tests have been replaced by PCR, which has been proven to be more sensitive. However, some difficulties must be addressed to overcome unequal results due to sample volume, DNA extraction protocol or *T. cruz*i region of amplification (Junqueira et al., 1996; Virreira et al., 2003). For this reason, more conserved *T. cruzi* regions have been targeted, such as the satellite DNA and the variable region of kinetoplast DNA (kDNA) mini-circles (Schijman et al., 2011; Ramirez et al., 2015).

The introduction of qPCR has significantly improved molecular biology techniques. Automatization and standardization have allowed for the quantification of *T. cruzi* parasitic loads (Qvarnstrom et al., 2012).

Several efforts have been made to improve the sensitivity and specificity of qPCR. **Table 1** provides a few examples of such research work.

**Table 1:** Characteristics of some improved qPCR protocols for *T. cruzi* detection.

Study	Methodology	Molecular target	Achievement
Piron et al., 2007	Standardized in-house TaqMan qPCR	T. cruzi satellite DNA	Reduced the risk of carry-over contamination
Ramírez et al., 2015	Commercial qPCR	T. cruzi satellite DNA	No differences were found between parasite loads of asymptomatic and symptomatic chronic patients
Benatar et al., 2021	Commercial real-time PCR	T. cruzi satellite DNA	In congenital diagnosis, it was shown that its sensitivity was twice that of micro hematocritic
Sulleiro et al., 2020	Standardized in house TaqMan qPCR	T. cruzi satellite DNA	Positivity in 42% of untreated chronic Chagas patients
Kann et al., 2020	Newly developed one (NOD-PCR) Standardized in house TaqMan qPCR	T. cruzi mini-circle DNA T. cruzi mini-satellite DNA T. cruzi small subunit ribosomal RNA	kDNA-PCR showed 77.3% false positive rate, cross reacting with T. rangeli. Mini-satellite showed a false negative rate of 79.5% and 18S small subunit ribosomal of 98.5%.

Digital droplet PCR is a technique in which the amplification reaction is conducted for individual nucleic acid molecules in thousands of independent PCR reactions, previously divided into droplets from a sample. The advantage of ddPCR is its ability to provide absolute quantification without the need for a standard curve (Liu et al., 2023). However, it has some limitations, including the high cost of instruments, the requirement for well-trained personnel, and a tendency to yield false-positive results. When it comes to detecting T. cruzi DNA in patients’ blood samples, ddPCR does not outperform qPCR, with a sensitivity of 1 parasite/mL compared to 0.46 parasite/mL in qPCR (Ramírez et al., 2018). The LAMP-PCR protocol for T. cruzi DNA amplification requires only one temperature for the reaction to occur, thanks to Bacillus stearothermophilus. It employs colorimetric or fluorescent dyes for *in situ* detection (Alves, 2020). LAMP has demonstrated high sensitivity, cost-effectiveness, and speed. However, it has raised some concerns due to its propensity for high levels of non-specific amplification (Shrestha et al., 2023). To address these limitations, Argentinian researchers have introduced additional steps in the protocol, including more stringent sample preparation and specific kits. Schijman’s group found that their T. cruzi Lamp kit was as sensitive as qPCR (Besuschio et al., 2020; Muñoz-Calderón et al., 2022).

## PCR in congenital Chagas disease

Early diagnosis and care are essential in congenital Chagas disease. Diagnosis in the early stages leads to the best outcomes for therapeutic success. However, this is challenging in the first months of life due to the transfer of maternal antibodies (Carlier and Truyens, 2015; Pecoul et al., 2016).

Early *T. cruzi* detection by qPCR can provide more accurate estimations of congenital cases. This could improve the early detection of cases, providing more accurate records on the number of infants born to Chagas disease mothers in endemic and non-endemic countries and allowing better estimation of case numbers. Early qPCR diagnosis tests have been done by Benatar et al. (2021) (**Table 1**) however they recognized that improvements need to be made. A year earlier, the same Schijman group used the LAMP-PCR test to analyze 13 congenital Chagas disease individuals and found that LAMP-PCR was sensitive and specific, comparable to qPCR (Besuschio et al., 2020). In a meta-analysis by Candia-Puma et al. (2022), it was found that qPCR is the most effective among molecular diagnostic tools, particularly in acute cases.

## PCR in chronic Chagas disease

Efforts have been made over the years to establish a standardized PCR protocol for monitoring the treatment of chronic patients, as serology alone is not accurate enough to validate treatment efficacy. In adults, antibodies against *T. cruzi* can remain detectable for six months to several years after treatment. Sulleiro et al. (2020) detected the presence of *T. cruzi* by qPCR in 42% of untreated chronic patients, with almost 55% of a subgroup of them showing intermittent parasitemia.

However, PCR negativity does not guarantee that the infection has been cured. Although treatment can demonstrate excellent effectiveness in eliminating blood-stage parasites, its capacity to target tissue forms remains uncertain (Simón et al., 2020).

There is still no consensus about the usefulness of PCR as a predictive marker of disease progression. Sulleiro et al. (2020) demonstrated that a positive qPCR is not necessarily associated with visceral abnormalities. However, Sabino et al. (2015) observed that a positive qPCR is linked to Chagas cardiomyopathy and disease severity, contradicting the findings of a smaller study by Norman et al. (2011).

As shown in **Table 1** more specific qPCR protocols have been developed; however, these tests are still recommended to be used in combination with serological tests, which could significantly improve Chagas disease treatment. This combination of tests can not only be useful for therapy indication, but also for monitoring, and control, as well as for surveillance of *T. cruzi* transmitters and control.

In 2022, Candia-Puma et al. (2022) performed a meta-analysis over the last 30 years. They observed that PCR and qPCR are not as good as the ELISA test, which proved to be the best diagnostic tool in acute and chronic Chagas disease. When they analyzed the molecular techniques, they found that these techniques have not been standardized. Despite its analytical validation, qPCR remains to be clinically validated to determine its practical usefulness (Duffy et al., 2013). Recent findings by Muñoz-Calderon et al. (2022) have shown promising results for LAMP-PCR. Even with a small sample size, they suggested that LAMP could be used as indicator of treatment failure.

## PCR in non-humans

In endemic areas, dogs and cats are considered as good indicators of potential active *T. cruzi* transmission. In the acute phase, Curtis-Robles et al. (2017) proposed the use of molecular methods to confirm infection. Additionally, molecular techniques could be useful for monitoring parasitemia during drug treatment of Chagas disease in dogs (Lana et al., 1991). However, in the chronic phase, dogs and cats generally show low and intermittent parasitemia (Eloy and Lucheis, 2009), which diminishes all diagnostic methodologies. This issue has also been found in animals in captivity, in 2000, Ndao and colleagues conducted a study involving a colony of captive New World monkeys (Ndao et al., 2000). Their research revealed an interesting phenomenon: among the monkeys initially tested negative for *T. cruzi* using PCR, a subsequent round of testing showed that a small subset of these monkeys (n=5) became positive on both smear and PCR test. This observation raised concerns regarding the possibility of false negatives.

## Discussion

Due to the intermittent nature of parasitemia in the chronic stage, it is difficult to determine the best time to obtain accurate results. Other factors to be considered are the strain of the parasite and the clinical variability, which have been attributed to the high genetic diversity and multiclonality of natural populations of *T. cruzi* (Macedo and Pena, 1998). Depending on the geographical origin of the strain and the source of infection, PCR values can vary, as several authors have published. The behavior of the strain is an important factor since the pattern of the release of the infective forms into the bloodstream is not well established. The lineage of the parasite must also be considered. *T. cruzi* populations show high genetic diversity and are classified into six Discrete Typing Units (DTUs) named TcI to TcVI (Zingales et al., 2012). The vast regional diversity and the course of chronic infection might reflect complex interactions between the genetic variability of *T. cruzi* strains, host immunogenetics, and eco-epidemiological characteristics (Moreira et al., 2013).

Given the fluctuating levels of parasitemia observed in individuals with chronic Chagas disease, it might be useful to perform repeated examinations with blood taken at different times using reliable qPCR kits. However, this can be challenging with a limited budget (Seiringer et al., 2017).

Currently, it is still recommended that PCR and qPCR be validated with a serological test. The robustness of immunological techniques has been well established (Ferrer et al., 2013), with ELISA being widely recognized for its performance (Candia-Puma et al., 2022).

However, qPCR is not exempt from limitations such as a higher cost of consumables compared to conventional methods. It requires a thermal cycler coupled with an optical reading system to allow for interpretation and a high level of technical skill. In general, molecular techniques require expensive resources and equipment. While LAMP is a promising technique, it requires further testing by other research groups in endemic settings to assess accessibility, affordability, accuracy, and sensitivity. There is a need for more rapid tests that do not sacrifice sensitivity and can be used in both clinical settings and resource-poor field settings. Research efforts should focus on the development of new diagnostic methods including serological, molecular, and proteomics approaches.

It is important to continue improving molecular tools with high-throughput instrumentation to provide more reliable and accurate results. However, affordability is essential in the neglected disease field. Simple technology and temperature-resistant reagents are mandatory. Techniques that can be implemented in the field without requiring sophisticated equipment and expensive reagents are needed. Identifying biomarkers for simple, easy-to-use tests is crucial. While some candidates have emerged, substantial efforts are still required to develop these kits and make them accessible in the field.

## Author contributions

NB-L: Conceptualization, Formal analysis, Writing – original draft, Writing – review & editing. MN: Conceptualization, Formal analysis, Project administration, Writing – review & editing.

## References

[B1] Alonso-Padilla J. Cortés-Serra N. Pinazo M. J. Bottazzi M. E. Abril M. Barreira F. . (2019). Strategies to enhance access to diagnosis and treatment for Chagas disease patients in Latin America. Expert Rev. Anti Infect. Ther. 17, 145–157. doi: 10.1080/14787210.2019.1577731 30712412

[B2] Alves G. Z. D. (2020). PCR and Infectious diseases. IntechOpen. doi: 10.5772/intechopen.85630

[B3] Benatar A. F. Danesi E. Besuschio S. A. Bortolotti S. Cafferata M. L. Ramirez J. C. . (2021). Prospective multicenter evaluation of real time PCR Kit prototype for early diagnosis of congenital Chagas disease. EBioMedicine. 69, 103450. doi: 10.1016/j.ebiom.2021.103450 34186488 PMC8243352

[B4] Besuschio S. A. Picado A. Muñoz-Calderón A. Wehrendt D. P. Fernández M. Benatar A. . (2020). *Trypanosoma cruzi* loop-mediated isothermal amplification (Trypanosoma cruzi Loopamp) kit for detection of congenital, acute and Chagas disease reactivation. PloS Negl. Trop. Dis. 14, e0008402. doi: 10.1371/journal.pntd.0008402 32797041 PMC7458301

[B5] Candia-Puma M. A. Machaca-Luque L. Y. Roque-Pumahuanca B. M. Galdino A. S. Giunchetti R. C. Coelho E. A. F. . (2022). Accuracy of diagnostic tests for the detection of chagas disease: a systematic review and meta-analysis. Diagnostics (Basel). 12, 2752. doi: 10.3390/diagnostics12112752 36359595 PMC9689806

[B6] Carlier Y. Truyens C. (2015). Congenital Chagas disease as an ecological model of interactions between *Trypanosoma cruzi* parasites, pregnant women, placenta and fetuses. Acta Trop. 151, 103–115. doi: 10.1016/j.actatropica.2015.07.016 26293886

[B7] Curtis-Robles R. Zecca I. B. Roman-Cruz V. Carbajal E. S. Auckland L. D. Flores I. . (2017). *Trypanosoma cruzi* (Agent of chagas disease) in sympatric human and dog populations in "Colonias" of the lower rio grande valley of Texas. Am. J. Trop. Med. Hyg. 96, 805–814. doi: 10.4269/ajtmh.16-0789 28167589 PMC5392625

[B8] Duffy T. Cura C. I. Ramirez J. C. Abate T. Cayo N. M. Parrado R. . (2013). Analytical performance of a multiplex Real-Time PCR assay using TaqMan probes for quantification of *Trypanosoma cruzi* satellite DNA in blood samples. PloS Negl. Trop. Dis. 1, e2000. doi: 10.1371/journal.pntd.0002000 PMC3547845 23350002

[B9] Echavarría N. G. Echeverría L. E. Stewart M. Gallego C. Saldarriaga C. (2021). Chagas disease: chronic chagas cardiomyopathy. Curr. Probl Cardiol. 46, 100507. doi: 10.1016/j.cpcardiol.2019.100507 31983471

[B10] Eloy L. J. Lucheis S. B. (2009). Canine trypanosomiasis: etiology of infection and implications for public health. J. Venom Anim. Toxins incl Trop. Dis. 15, 589–611. doi: 10.1590/S1678-91992009000400002

[B11] Ferrer E. Lares M. Viettri M. Medina M. (2013). Comparación entre técnicas inmunológicas y moleculares para el diagnóstico de la enfermedad de Chagas [Comparison between immunological and molecular techniques for the diagnosis of Chagas disease]. Enferm Infecc Microbiol. Clin. 5, 277–282. doi: 10.1016/j.eimc.2012.09.007 23102613

[B12] Junqueira A. C. Chiari E. Wincker P. (1996). Comparison of the polymerase chain reaction with two classical parasitological methods for the diagnosis of Chagas disease in an endemic region of north-eastern Brazil. Trans. R Soc. Trop. Med. Hyg. 90, 129–132. doi: 10.1016/s0035-9203(96)90111-x 8761570

[B13] Kann S. Kunz M. Hansen J. Sievertsen J. Crespo J. J. Loperena A. . (2020). Chagas disease: detection of *trypanosoma cruzi* by a new, high-specific real time PCR. J. Clin. Med. 9, 1517–1531 doi: 10.3390/jcm9051517 PMC7291166 32443464

[B14] Lana M. Vieira L. M. MaChado-Coelho G. L. Chiari E. Veloso V. M. Tafuri W. L. (1991). Humoral immune response in dogs experimentally infected with *Trypanosoma cruzi* . Mem Inst Oswaldo Cruz 86, 471–473. doi: 10.1590/s0074-02761991000400019 1842441

[B15] Liu Q. Jin X. Cheng J. Zhou H. Zhang Y. Dai Y. (2023). Advances in the application of molecular diagnostic techniques for the detection of infectious disease pathogens (Review). Mol. Med. Rep. 27, 104. doi: 10.3892/mmr.2023.12991 37026505 PMC10086565

[B16] Macedo A. M. Pena S. D. (1998). Genetic variability of trypanosoma cruzi:Implications for the pathogenesis of chagas disease. Parasitol. Today 14, 119–124. doi: 10.1016/s0169-4758(97)01179-4 17040719

[B17] Moreira O. C. Ramírez J. D. Velázquez E. Melo M. F. Lima-Ferreira C. Guhl F. . (2013). Towards the establishment of a consensus real-time qPCR to monitor *Trypanosoma cruzi* parasitemia in patients with chronic Chagas disease cardiomyopathy: a substudy from the BENEFIT trial. Acta Trop. 125, 23–31. doi: 10.1016/j.actatropica.2012.08.020 22982466

[B18] Muñoz-Calderón A. A. Besuschio S. A. Wong S. Fernández M. García Cáceres L. J. Giorgio P. . (2022). Loop-mediated isothermal amplification of *trypanosoma cruzi* DNA for point-of-care follow-up of anti-parasitic treatment of chagas disease. Microorganisms. 10, 909. doi: 10.3390/microorganisms10050909 35630354 PMC9142941

[B19] Ndao M. Kelly N. Normandin D. MacLean D. J. Whiteman A. Kokoskin E. . (2000). *Trypanosoma cruzi* infection of squirrel monkeys: Comparison of blood smear examination, commercial enzyme-linked immunosorbent assay, and polymerase chain reaction analysis as screening tests for evaluation of monkey-related injuries. Comp. Med. 50, 658–665. 11200574

[B20] Norman F. F. Pérez-Ayala A. Pérez-Molina J. A. Flores-Chavez M. Cañavate C. López-Vélez R. (2011). Lack of association between blood-based detection of *Trypanosoma cruzi* DNA and cardiac involvement in a non-endemic area. Ann. Trop. Med. Parasitol. 105, 425–430. doi: 10.1179/1364859411Y.0000000033 22117851 PMC4100305

[B21] Pecoul B. Batista C. Stobbaerts E. Ribeiro I. Vilasanjuan R. Gascon J. . (2016). The BENEFIT trial: where do we go from here? PloS Negl. Trop. Dis. 10, e0004343. doi: 10.1371/journal.pntd.0004343 26913759 PMC4767872

[B22] Piron M. Fisa R. Casamitjana N. López-Chejade P. Puig L. Vergés M. . (2007). Development of a real-time PCR assay for *Trypanosoma cruzi* detection in blood samples. Acta Trop. 103, 195–200. doi: 10.1016/j.actatropica.2007.05.019 17662227

[B23] Qvarnstrom Y. Schijman A. G. Veron V. Aznar C. Steurer F. da Silva A. J. (2012). Sensitive and specific detection of *Trypanosoma cruzi* DNA in clinical specimens using a multi-target real-time PCR approach. PloS Negl. Trop. Dis. 6, e1689. doi: 10.1371/journal.pntd.0001689 22802973 PMC3389027

[B24] Ramírez J. C. Cura C. I. da Cruz Moreira O. Lages-Silva E. Juiz N. Velázquez E. . (2015). Analytical validation of quantitative real-time PCR methods for quantification of *trypanosoma cruzi* DNA in blood samples from chagas disease patients. J. Mol. Diagn. 17, 605–615. doi: 10.1016/j.jmoldx.2015.04.010 26320872 PMC4698797

[B25] Ramírez J. D. Herrera G. Hernández C. Cruz-Saavedra L. Muñoz M. Flórez C. . (2018). Evaluation of the analytical and diagnostic performance of a digital droplet polymerase chain reaction (ddPCR) assay to detect Trypanosoma cruzi DNA in blood samples. PloS Negl. Trop. Dis. 12, e0007063. doi: 10.1371/journal.pntd.0007063 30586355 PMC6324824

[B26] Rangel-Gamboa L. López-García L. Moreno-Sánchez F. Hoyo-Ulloa I. Vega-Mémije M. E. Mendoza-Bazán N. . (2019). *Trypanosoma cruzi* infection associated with atypical clinical manifestation during the acute phase of the Chagas disease. Parasit Vectors. 12, 506–512. doi: 10.1186/s13071-019-3766-3 31666114 PMC6822409

[B27] Sabino E. C. Ribeiro A. L. Lee T. H. Oliveira C. L. Carneiro-Proietti A. B. Antunes A. P. . (2015). Chagas Study Group of the NHLBI Retrovirus Epidemiology Donor Study-II, International Component. Detection of *Trypanosoma cruzi* DNA in blood by PCR is associated with Chagas cardiomyopathy and disease severity. Eur. J. Heart Fail. 17, 416–423. doi: 10.1002/ejhf.220 25678239 PMC5665686

[B28] Schijman A. G. Bisio M. Orellana L. Sued M. Duffy T. Mejia Jaramillo A. M. . (2011). International study to evaluate PCR methods for detection of *Trypanosoma cruz*i DNA in blood samples from Chagas disease patients. PloS Negl. Trop. Dis. 5, e931. doi: 10.1371/journal.pntd.0000931 21264349 PMC3019106

[B29] Seiringer P. Pritsch M. Flores-Chavez M. Marchisio E. Helfrich K. Mengele C. . (2017). Comparison of four PCR methods for efficient detection of *Trypanosoma cruzi* in routine diagnostics. Diagn. Microbiol. Infect. Dis. 88, 225–232. doi: 10.1016/j.diagmicrobio.2017.04.003 28456430

[B30] Shrestha K. Kim S. Han J. Florez G. M. Truong H. Hoang T. . (2023). Mobile efficient diagnostics of infectious diseases via on-chip RT-qPCR: MEDIC-PCR. Adv. Sci. (Weinh) 10, e2302072. doi: 10.1002/advs.202302072 37587764 PMC10558658

[B31] Simón M. Iborra M. A. Carrilero B. Segovia M. (2020). What is the role of real time PCR in the follow up of patients with chronic Chagas' disease? Enferm Infecc Microbiol. Clin. (Engl Ed). 38, 353–355. doi: 10.1016/j.eimc.2020.07.002 32912656

[B32] Sulleiro E. Salvador F. Martínez de Salazar P. Silgado A. Serre-Delcor N. Oliveira I. . (2020). Contributions of molecular techniques in the chronic phase of Chagas disease in the absence of treatment. Enferm Infecc Microbiol. Clin. (Engl Ed). 38, 356–360. doi: 10.1016/j.eimc.2020.01.003 32087978

[B33] Virreira M. Torrico F. Truyens C. Alonso-Vega C. Solano M. Carlier Y. . (2003). Comparison of polymerase chain reaction methods for reliable and easy detection of congenital *Trypanosoma cruzi* infection. Am. J. Trop. Med. Hyg. 68, 574–582. doi: 10.4269/ajtmh.2003.68.574 12812349

[B34] World Health Organization (WHO) . (2020). Chagas disease (American trypanosomiasis). Available at: https://www.who.int/health-topics/chagas-disease#tab=tab_1 (Accessed 15 May 2023).

[B35] Zingales B. Miles M. A. Campbell D. ,. A. Tibayrenc M. Macedo A. M. Teixeira M. M. . (2012). The revised Trypanosoma cruzi subspecific nomenclature: rationale, epidemiological relevance and research applications. Infect. Genet. Evol. 12, 240–253. doi: 10.1016/j.meegid.2011.12.009 22226704

